# Efficacy and toxicity of chemoradiation in patients with anal cancer - a retrospective analysis

**DOI:** 10.1186/1748-717X-9-113

**Published:** 2014-05-13

**Authors:** Stefan Alexander Koerber, Alla Slynko, Matthias F Haefner, David Krug, Clara Schoneweg, Kerstin Kessel, Annette Kopp-Schneider, Klaus Herfarth, Juergen Debus, Florian Sterzing

**Affiliations:** 1 Department of Radiation Oncology, University Hospital, Heidelberg, Germany; 2 Clinical Cooperation Unit Radiation Oncology, German Cancer Research Center, Heidelberg, Germany; 3 Institute of Biostatistics, German Cancer Research Center, Heidelberg, Germany

**Keywords:** Anal [canal] cancer, Radiochemotherapy, Toxicity, IMRT, 3D-CRT, Radiation

## Abstract

**Background:**

Concurrent chemotherapy and radiation therapy is the preferred standard of care for patients with anal cancer. Several studies have suggested a benefit of intensity-modulated radiation therapy (IMRT) compared with 3D-conformal radiation (3D-CRT) regarding acute toxicity. This study evaluates outcome and toxicity of patients undergoing IMRT/Tomotherapy or 3D-CRT at our institution.

**Methods:**

A cohort of 105 anal cancer patients was treated with chemoradiation or radiation alone (16.2%) between January 2000 and December 2011. 37 patients received 3D-CRT while 68 patients were treated with IMRT. Follow-up exams were performed every 3 to 6 months for a minimum of 3 years and then annually.

**Results:**

Median follow-up was 41.4 months (2.8 – 158.4). Overall survival (OS), Progression-free survival (PFS) and local control (LC) at 3 years was 70.3%, 66.5%, 78.3% in the 3D-CRT group and 82.9%, 66.5%, 75.3% in the IMRT group without statistically significant difference. 3-year Colostomy-free survival (CFS) was 85.7% in the IMRT/Tomotherapy group and 91.8% in the 3D-CRT group (p = 0.48). No grade 4 toxicity was found in both groups. Severe (G2/3) acute skin toxicity (94.6% vs. 63.2%; p < 0.001) and acute gastrointestinal toxicity rate (67.6% vs. 47.1%; p = 0.03) was significantly higher with 3D-CRT compared to IMRT/Tomotherapy.

**Conclusion:**

The use of IMRT can reduce acute severe side effects of the skin and gastrointestinal tract but did not demonstrate improved results regarding OS, PFS, LC and CFS.

## Background

With age-standardised incidence rates between 1 and 2 per 100000 per year anal cancer is the less frequent GI tumours, but several studies have shown an increasing incidence over the past years [1-4]. The long-term update of the Radiation Therapy Oncology Group (RTOG) 98–11 trial confirmed the established concurrent chemoradiation (CCR) with 5-fluorouracil (5-FU) plus mitomycin (MMC) as the preferred standard of care. The 5 year disease-free survival (DFS) of 67.8% and 5 year overall survival (OS) of 78.3% were achieved at the cost of a high incidence of grade 3 and 4 toxicity [5]. These side effects were in part attributed to the use of older radiation techniques (RT) such as nonconformal AP/PA radiotherapy, which leads to a high dose exposure in normal tissues such as genitalia or small bowel. Today, many oncological centres use modern techniques like step-and-shoot intensity-modulated radiation therapy (IMRT) or Tomotherapy, as recommended by the National Comprehensive Cancer Network (NCCN), for the treatment of patients with anal cancer [6]. A couple of institutions reported, that treatment-related side effects could be dramatically reduced while receiving a good outcome using IMRT for anal cancer [7,8].

At the department of radiation oncology, University Hospital Heidelberg, and at the department of radiation oncology at the German Cancer Research Center, Heidelberg, IMRT for radiation therapy for anal cancer patients was introduced during 2003–2004. This retrospective analysis reports on the efficacy and toxicity of anal cancer patients undergoing 3D-conformal radiation (3D-CRT) or IMRT/Tomotherapy at our institution.

## Methods

### Patient and tumor characteristics

From January 2000 to December 2011 a total of 105 anal cancer patients were treated with radiotherapy or radiochemotherapy at the department of radiation oncology, Heidelberg. Patient and tumour characteristics are shown in Table 1. 37 patients with a median age of 61.0 years (range 35 – 94 years) received a 3D-CRT while 68 patients with a median age of 54 years (range 22 – 86 years) were treated with IMRT/Tomotherapy. Women were affected more frequently than men (80.9% vs. 19.1% in the IMRT/Tomotherapy group; 97.3% vs. 2.7% in the 3D-CRT group). In the majority of cases histological examination showed squamous cell carcinoma. About 50% of the patients had a tumour size from 2 – 5 cm, typically without any distant metastases (97.1% vs. 100%). 13 patients (35.1%) of the 3D-CRT group and 12 patients (17.6%) of the IMRT/Tomotherapy group underwent local excision before chemoradiation (Rx: 16%; R0: 16%; R1: 60%; R2: 8%). 21.6% in the 3D-CRT group and 5.9% in the IMRT/Tomotherapy group received colostomies immediately following the initial diagnosis before starting chemoradiation (time range: 0.1 – 2.6 months). The most common indications for colostomy included protective reasons and clinically manifest subileus.

**Table 1:** Patient and tumour characteristics according to the type of radiotherapy (3D-CRT vs. IMRT/Tomotherapy)

	**3D-CRT**	**IMRT/Tomotherapy**
**Total number of patients**	37 (35.2%)	68 (64.8%)
**Age [years]**		
Median	61	54
Range	35 - 94	22 - 86
**Sex**		
Male	1 (2.7%)	13 (19.1%)
Female	36 (97.3%)	55 (80.9%)
**T stage**		
T1	9 (24.3%)	11 (16.2%)
T2	18 (48.7%)	36 (52.9%)
T3	6 (16.2%)	13 (19.1%)
T4	4 (10.8%)	8 (11.8%)
**N stage**		
N0	29 (78.4%)	50 (73.5%)
N1	1 (2.7%)	8 (11.8%)
N2	5 (13.5%)	4 (5.9%)
N3	2 (5.4%)	6 (8.8%)
**M stage**		
M0	37 (100%)	66 (97.1%)
M1	0 (0%)	2 (2.9%)
**Histology**		
Squamous cell	35 (94.6%)	65 (95.6%)
Other (small cell, basaloid)	2 (5.4%)	3 (4.4%)

### Radiochemotherapy

**Figure 1:**
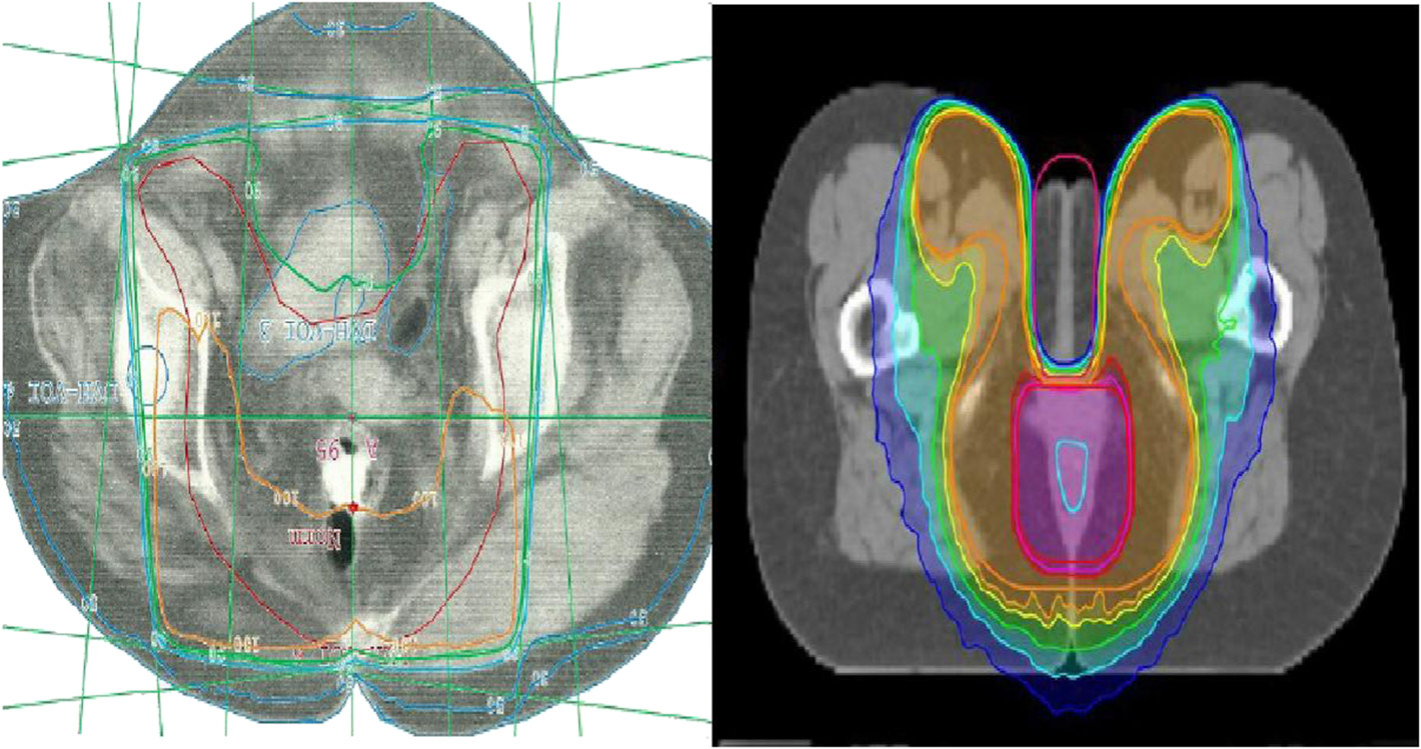
Dose distribution on planning CT with 3D-CRT and IMRT for a patient with anal cancer [from left to right].

Patients were simulated in supine position before radiotherapy using CT-scans and knee support for immobilisation. 3D-CRT was performed using a standard three- to five-field box including the pelvis from promontorium to the perianal region. The first radiation sequence (median 45.0 Gray (Gy); range 36.0 – 50.4 Gy) involved the macroscopic tumour, bilateral iliac, inguinal and pararectal lymph nodes. For the second radiation sequence (to a median cumulative dose 54.0 Gy; range 45.0 – 61.1 Gy) the volume was radically reduced to include the macroscopic tumour and the involved lymph nodes. For superficial lesions an additional boost with electrons was performed (35.1%). 14 patients received a boost with electrons and photons, the other patients (10; 27.0%) received only a photon boost. Daily fraction dose was 1.8 Gy in the first and second radiaton sequence. For IMRT/Tomotherapy anatomical structures and target volumes were countoured based on MRI and CT images. Gross tumour volume (GTV) was defined as visible tumour mass or involved node(s) on imaging. Elective nodal volumes including mesorectum, presacral space, ischiorectal fossa, inguinal/obturator/iliac (internal and external) lymph nodes were contoured. By combining GTV, the complete anal canal and both anal sphincters with 20 mm margins the clinical target volume (CTV) was obtained. For planning target volume (PTV) CTV was enlarged by 10 mm (Figure 1). IMRT/Tomotherapy was performed using daily image guidance. Median radiation dose of elective nodes was 45.0 Gy (range 36.0 – 50.4 Gy). Radiotherapy delivered a dose of up to 63.2 Gy with a median dose of 54.5 Gy to gross tumour and involved nodes by using simultaneous integrated boost technique. Radiotherapy was done five days a week (once daily) in 1.8 Gy/2.2 Gy daily fractions respectively.

During chemoradiation women received vaginal tampons and cones to spare the anterior vaginal wall. Vaginal dilators were offered women after therapy to prevent vaginal stenosis. Of the 105 patients, 79 received concomitant radiochemotherapy with 5-fluorouracil 1000 mg/m^2^ body surface (typically days 1–5 and 29–33) plus mitomycin 10 mg/m^2^ body surface (days 1 and 29). A discontinuation of therapy after the first chemotherapy cycle was necessary because of fulminant side effects (e.g. thrombocytopenia) for 9 patients. 3 patients obtained only 5-FU and 2 cisplatin plus 5-FU. Patients with relevant comorbidities (e.g. cardiac conditions) and/or poor Karnofsky performance score (16) received only radiotherapy, 5 patients received concomitant cetuximab weekly in an individual setting.

### Clinical course and follow-up

During therapy patients were examined at least once a week (including blood tests and physical examination). After radiotherapy or radiochemotherapy follow-up exams were performed every 3 to 6 months for a minimum of 3 years and then annually. Routine follow-up consisted of physical examination, imaging (MRI or CT), rectoscopy and biopsy for abnormal findings. Treatment response was determined based upon Response Evaluation Criteria In Solid Tumours (RECIST), version 1.1. Acute and long-term side effects were scored according to Common Terminology Criteria for Adverse Effects (CTCAE), version 4.0. Late toxicity was defined as adverse effects apparent 6 months after therapy.

### Statistical analysis

All survival end points were calculated starting from the first diagnosis date. OS was then defined as the time to death. LC was defined as the time to locally progressive disease of the primary tumour or regional lymph nodes. PFS and CFS were defined as the time to progressive disease/time to colostomy or death. All patients who did not experience the event of interest were censored at the last follow-up date. The Kaplan-Meier method was utilized to estimate OS, PFS, CFS and LC for various group partitions. Univariate survival time comparisons were performed using the log-rank test. Multivariate analyses, where possible, were performed using Cox regression. Univariate analysis of acute toxicity in the 3D-CRT and IMRT/Tomotherapy groups was performed using the exact Fisher test. The statistical analysis was performed using R version 3.0.2. This retrospective analysis was approved by local ethics committee Heidelberg.

## Results

The cohort of 105 patients treated by IMRT/Tomotherapy or 3D-CRT showed no significant differences regarding patient and tumour characteristics except for a higher proportion of women in the 3D-CRT group.

### Tumour response and outcome

**Figure 2:**
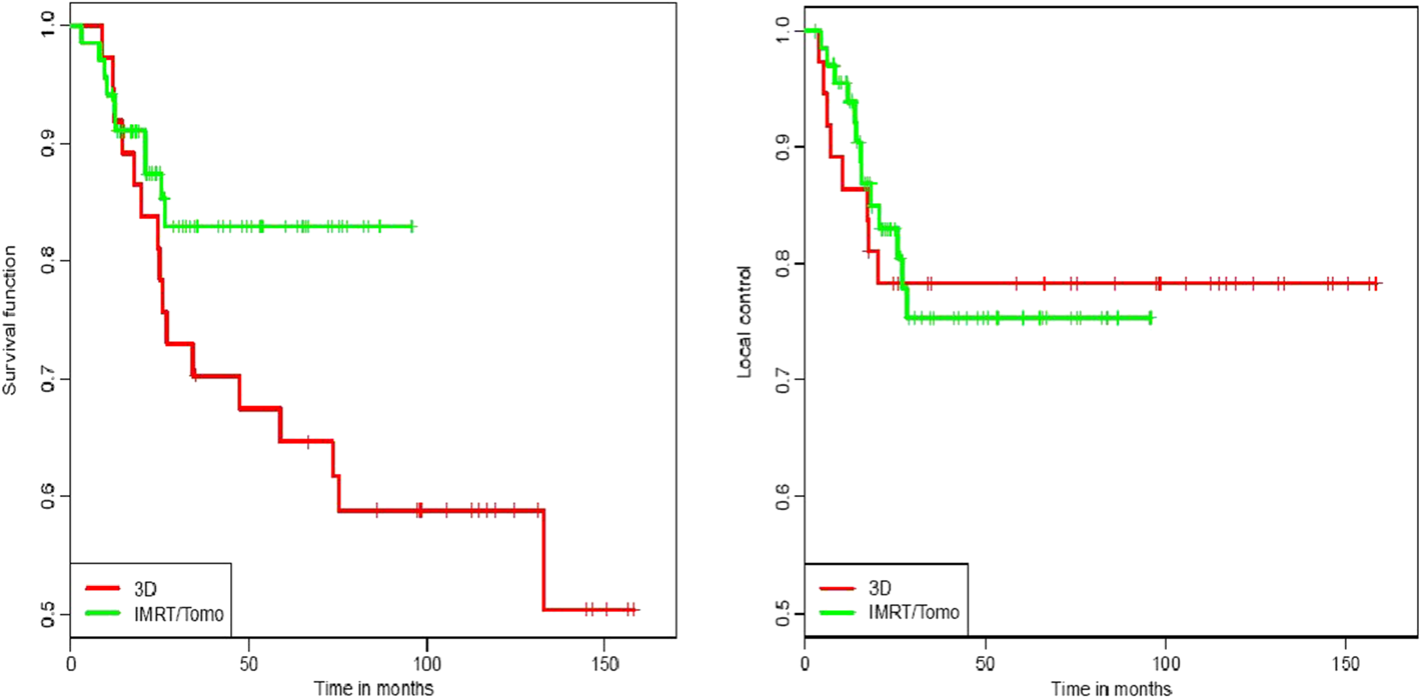
Kaplan-Meier estimates of OS and LC for 3D-CRT and IMRT/Tomotherapy group [from left to right].

**Figure 3:**
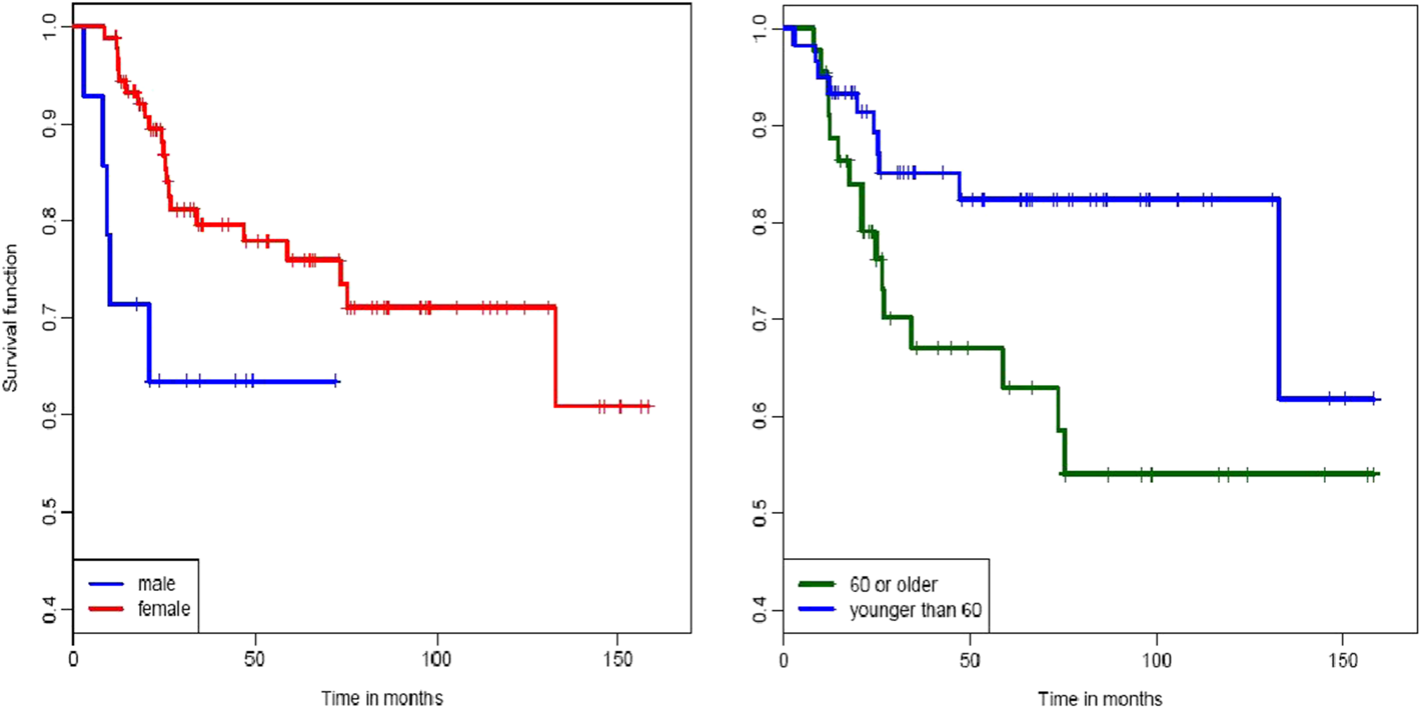
Kaplan-Meier estimates of OS according to gender and age (cut-off 60 years) [from left to right].

Median follow-up for the whole cohort was 41.4 months (2.8 - 158.4), for the 3D-CRT group 97.5 months and for the IMRT group 30.8 months. Concerning the follow-up examination imaging showed 64 (60.9%) complete remissions (CR), 5 (4.8%) partial remissions (PR) and 5 (4.8%) patients with stable disease (SD) in both treatment groups. 12 patients in the 3D-CRT group and 21 patients in the IMRT/Tomotherapy group (total 33, 31.4%) developed progressive disease (PD), 8/13 of them a local progression. 3/7 patients relapsed with distant metastases, 1/1 presented with systemic and local relapse. In these cases patients underwent further therapy, e.g. abdominoperineal resection or palliative chemotherapy. One patient was lost to follow-up, one patient died during chemoradiation because of an unknown Fanconi anaemia. The 2-year and 3-year OS for the whole cohort was 86.0% and 77.4% respectively. Regarding the two different radiation therapy groups, OS rate at 2 and 3 years was 83.8% and 70.3% in the 3D-CRT group and 87.4% and 82.9% in the IMRT/Tomotherapy group (Figure 2). There was a trend towards better survival in patients treated with IMRT/Tomotherapy, although this difference was not statistically significant (p = 0.07). Subgroup analysis of the entire cohort regarding gender and age showed a 2-year OS of 63.5% for male and 89.5% for female patients (p = 0.05) and a significantly better OS at 2 years for patients younger than 60 years (91.4% vs. 79.0%; p = 0.03) (Figure 3).

PFS rate at 2/3 years was 73.3%/66.5%, respectively. 2- and 3-year PFS rates of 72.9%/66.5% and 73.3%/66.5% were observed in the 3D-CRT and IMRT/Tomotherapy group (p = 0.88). 2- and 3-year LC showed no statistically significant difference between the two radiation therapy groups (p > 0.99), with a 78.3%/78.3% rate vs. 83.0%/75.3% rate in the 3D-CRT and IMRT/Tomotherapy group. CFS rate at 2 and 3 years was 89.6% and 87.9% for the whole cohort, 91.8%/91.8% in the 3D-CRT group and 88.6%/85.7% in the IMRT/Tomotherapy group (p = 0.48).

### Subgroup analysis for different treatment groups

**Figure 4:**
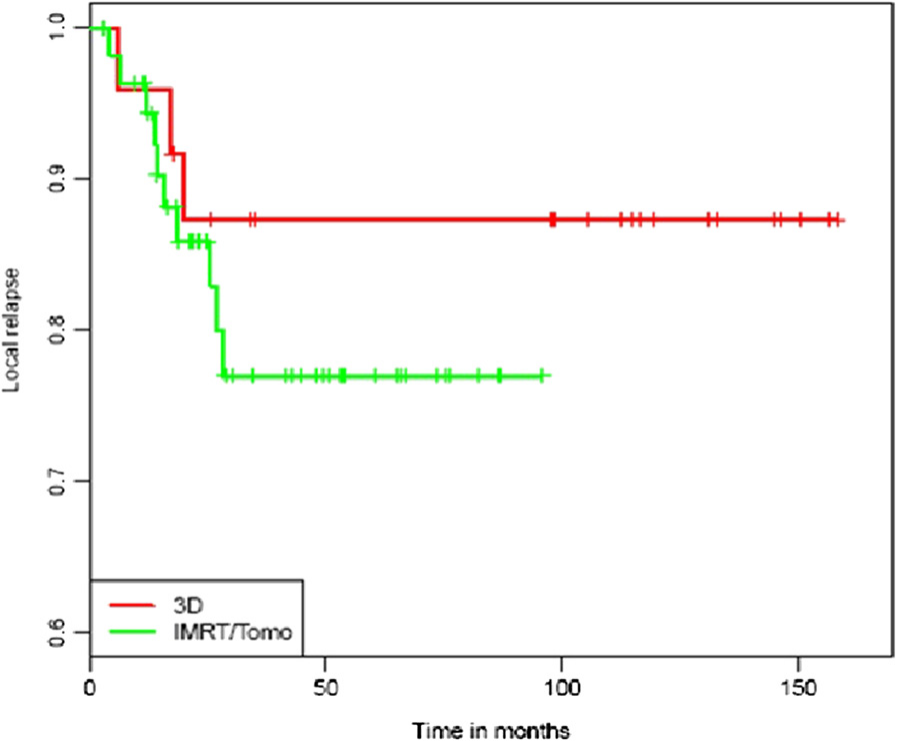
Kaplan-Meier estimates of LC in the standard chemoradiation group for 3D-CRT and IMRT/Tomotherapy.

Subgroup analysis was performed for all 79 patients receiving standard chemoradiation with 5-fluorouracil and mitomycin. No statistically significant difference could be found regarding OS and CFS for the 3D-CRT and IMRT/Tomotherapy group (p = 0.15 and p = 0.33) with 2-year OS rates of 87.5% vs. 90.1%. Despite an estimated 3-year PFS of 77.3% (3D-CRT) vs. 69.9% (IMRT/Tomotherapy) the values did not reach statistical difference (p = 0.36; Figure 4). A total of 20 patients (25.3%) developed progressive disease in this cohort (Table 2). 11 patients (13.9%) relapsed locally or within the regional lymph nodes whereas most of these recurrences occurred infield (90.0%). Systemic relapse was observed in 7 patients (8.9%), two patients developed local and systemic relapse. In most cases distant metastases occurred in liver or lung. There was no statistical difference in local or systemic relapse regarding both treatment groups (3D-CRT vs. IMRT/Tomotherapy). Median OS and PFS for the cohort of patients (5) receiving radiation and cetuximab were 26.8 and 15.5 months. Progressive disease was observed in 2 patients (40%). One patient developed local recurrence (infield), liver metastases occured in the other patient.

**Table 2:** Patterns of recurrence according to the type of radiotherapy (3D-CRT vs. IMRT/Tomotherapy)

	**3D-CRT**	**IMRT/Tomotherapy**
Total number of patients	24	55
Progressive disease (PD)	5 (20.8%)	15 (27.3%)
**Local recurrence**	2 (8.3%)	9 (16.4%)
Infield	2 (8.3%)	8 (14.6%)
Outfield	0	1 (1.8%)
**Systemic relapse**	2 (8.3%)	5 (9.1%)
**Local and systemic relapse**	1 (4.2%)	1 (1.8%)

Anal cancer was situated at the anal margin in five patients. One patient died during chemoradiation (unknown Fanconi anaemia), two patients were treated with radiotherapy alone. Median OS and PFS for this group were 24.1 and 15.7 months. Local recurrence was observed in two patients (50%) localized as an infield relapse.

### Influence on OS, PFS and LC

Different tumour and treatment characteristics (T-stage, technique of radiotherapy and concurrent chemotherapy) were tested as predictive factors in multivariate analysis. Tumour size (p = 0.04) and the use of chemotherapy (p = 0.03) had a significant influence on OS. For PFS and LC, only tumour size was a predictive factor (p = 0.03/0.004).

### Acute and late toxicity

In both therapy groups, no high-grade adverse events (G4) were found both for acute and late toxicity. No or minimal symptoms (G0/1) on the one hand, moderate and severe symptoms (G2/3) on the other hand were summarized according to limitation of Activities of Daily Living (ADL). Acute and late toxicity are listed in Table 3. 94.6% of 3D-CRT patients and 63.2% of IMRT/Tomotherapy patients experienced acute severe (G2/3) dermatologic toxicity (p < 0.001). The rate of acute severe toxicity of the gastrointestinal tract was 67.6% in the 3D-CRT group and 47.1% in the IMRT/Tomotherapy group (p = 0.03). Severe colpitis and dysuria occurred in 27% vs. 25% and 21.6% vs. 19.1% (3D-CRT vs. IMRT/Tomotherapy group) - without statistically significant differences between the considered two groups. G2 and G3 chronic toxicitiy regarding dysuria was observed for only 1 (2.7%) vs. 2 (2.9%) patients (3D-CRT vs. IMRT/Tomotherapy) undergoing chemoradiation. The rate of severe atrophy of vaginal mucosa was 16.2% in both treatment groups. A total of 4 (3.8%) patients developed faecal incontinence after chemoradiation, 2 (5.4%) in the 3D-CRT group (G2: 2 patients), 2 (2.9%) in the IMRT/Tomotherapy group (G1: 2 patients). Male patients didn’t develop G2 or G3 toxicity regarding potency. In all cases multivariate analysis has not been performed due to the small number of events.

**Table 3:** Acute and late toxicity according to the type of radiotherapy (3D-CRT vs. IMRT/Tomotherapy)

	**3D-CRT**		**IMRT/Tomotherapy**	
	**G0/G1**	**G2/3**	**G0/1**	**G2/3**
**Acute toxicity**				
Radiodermatitis	2 (5.4%)	35 (94.6%)	25 (36.8%)	43 (63.2%)
Urinary tract (dysuria)	29 (78.4%)	8 (21.6%)	55 (80.9%)	13 (19.1%)
Gastrointestinal (diarrhea)	12 (32.4%)	25 (67.6%)	36 (52.9%)	32 (47.1%)
Genital tract [*female*] (colpitis)	27 (73.0%)	10 (27.0%)	51 (75.0%)	17 (25.0%)
**Late toxicity**				
Urinary tract (dysuria)	36 (97.3%)	1 (2.7%)	66 (97.1%)	2 (2.9%)
Gastrointestinal (incontinence)	35 (94.6%)	2 (5.4%)	68 (100%)	0
Genital tract [*female*] (atrophy of vaginal mucosa)	31 (83.8%)	6 (16.2%)	57 (83.8%)	11 (16.2%)
Genital tract [*male*] (impotence)	37 (100%)	0	68 (100%)	0

## Discussion

For many years radiotherapy plus chemotherapy (5-FU and MMC) has represented the international standard of care for patients with anal carcinoma [9-11]. This leading treatment role has been confirmed by a 13-year follow-up showing only a small proportion of localregional relapse and cancer deaths undergoing a radiochemotherapy compared with RT alone [12]. Several studies suggest an equivalent if not better outcome with decreased toxicity by modern radiation techniques such as IMRT when compared to 3D-CRT [8,13,14]. The present analysis studied a large cohort of 105 patients with anal cancer treated with radiation therapy or chemoradiation at the department of radiation oncology, University Hospital Heidelberg and at the department of radiation oncology at the German Cancer Research Center, Heidelberg. The 3-year OS rate of 77.4% in our cohort was similar to that seen in comparable studies such as Bazan et al. with 72.0% [13]. Regarding 3D-CRT Dewas et al. reported an 81.1% 2-year OS [15] which is consistent with the 2-year OS rate of our cohort of 83.8%. In the present study 2-year OS of the IMRT/Tomotherapy group was 87.4%, respectively. Findings from other studies on IMRT showed 2-year OS ranging from 86.9% to 96.0% [15-17]. Even if no statistically significant overall survival difference between the two radiation technique groups was found (p = 0.07) as reported by Bazan et al. (3-year OS 88% vs. 52%; p < 0.01) [13], there seems to be a trend favoring IMRT/Tomotherapy. The lower OS in the 3D-CRT group observed in our study is multifactorial and could be explained by a tendency to an older population in the 3D-CRT cohort (median age 61 years vs. 54 years, p > 0.05). This fact and the associated larger proportion of comorbidities explain why patients in the IMRT group received more chemotherapy than 3D-CRT-patients (88.2% vs. 67.6%,, p = 0.02). In contrast, when only looking at the standard-chemoradiation cohort the trend towards a better OS in the IMRT/Tomotherapy group is much less pronounced.

It is interesting to note, that men showed a trend towards a worse OS (2-year 63.5% vs. 89.5%; p = 0.05) in multivariate analysis. Similar results could be found by the long-term update of RTOG 98–11 (p = 0.03) [5]. A significantly better OS at 2 years was observed regarding patients younger than 60 years (p = 0.03). Assuming that prevalence of human papillomavirus (HPV) infection in women decreases with increasing age [18], a relation between HPV status and outcome can be suggested similar to observations in other tumour entitites e.g. in oropharyngeal cancer [19]. Furthermore patients with cancer of the anal margin and patients receiving concurrent cetuximab showed worse median OS and PFS than the cohort of patients with anal canal tumours treated with standard chemoradiation (24.1 and 15.7 months, 26.8 and 15.5. months vs. 48.0 and 41.2 months). In almost half of the patients (50% and 40%) progressive disease was observed during follow-up, usually within the first two years. Even though interpretation is quite problematic due to small subgroups, especially the role of cetuximab should be discussed. A high surface expression of Epidermal Growth Factor Receptor (EGFR) and low rates of EGFR and k-ras mutations seem to enable the use of cetuximab for anal cancer treatment [20], but only a couple of studies describe a small benefit regarding palliative treatment of anal cancer patients [21,22]. In accordance with findings from the treatment of metastatic colorectal or head and neck cancer patients, Lukan et al. observed a PR in 3 of 7 patients with metastatic anal cancer and k-ras wildtype [23]. To the author’s knowledge no positive data exist regarding the use of cetuximab in a curative setting. One phase I trial and one phase II trial of patients with locally advanced anal cancer receiving 5-FU, cisplatin, cetuximab and radiotherapy reported inhomogeneous efficacy and a extremely high number of serious adverse effects (SAEs) [24,25]. Further studies in large population are required to identify the prognostic role of cetuximab and the influence of HPV and other biomarkers.

We found similar 3-year PFS (66.5% vs. 66.5%; p = 0.88) and LC (78.3% vs. 75.3%; p > 0.99) in the 3D-CRT group compared with the IMRT/Tomotherapy group. It is not completely understood, why 3-year PFS ended up being worse than those results presented by some other studies. PFS of 72.5% (3D-CRT) and 82.3% (IMRT) at 3 years has been reported by Chuong et al. e.g. [26]. Follow up in our cohort showed a relatively large proportion (31.4%) of PD compared with published data with values around 20% [15,16,27]. No difference could be found regarding patient, tumour or treatment characteristics except for a large number of tumour grading ≥ 3 (31.4%) in comparison with the literature. No statistically significant difference between the two radiation technique groups was found regarding CFS (p = 0.48). 3-year CFS rate of 87.9% in our cohort is comparable with findings from other studies showed 3-year CFS ranging from 86% to 91.3%/93.7% [13,26].

Several studies suggested decreased toxicity rates by using modern radiation techniques like IMRT [13,26]. These data were confirmed in our cohort considering both acute and late toxicity. In general no grade 4 acute or late adverse effects were observed. Severe (G2/3) dermatologic toxicity rate in the IMRT/Tomotherapy group was significantly lower than in the 3D-CRT group (63.2% vs. 94.6%; p < 0.001). Furthermore we recorded a significantly increased proportion of severe acute gastrointestinal toxicity in our cohort of 3D-CRT patients (67.6% vs. 47.1%; p = 0.03). Several studies generated similar results. While RTOG 98–11 showed an incidence of just under 50% for grade ≥ 3 skin toxicity using 2D-RT [28], Bazan et al. reported a significantly lower rate of grade 3 acute toxicity in the IMRT group compared with the 3D-CRT group (21% vs. 41%) [13]. In the same study grade ≥ 3 gastrointestinal toxicity was 29% and 7% in patients treated with 3D-CRT and IMRT [13]. No significant difference was observed regarding acute genitourinary toxicity in both groups. Although late adverse effects were present in our cohort, the number of events was small in general. 16.2% of the 3D-CRT and IMRT/Tomotherapy group developed moderate symptoms regarding to genital tract. Only one G3 genital toxicity in the 3D-CRT group and no G3 dysuria was observed. Similar results were reported by Kim et al. with no grade 3 or 4 late toxicities and an urinary incontinence rate of 2.7% [29]. 3 of 4 patients (75%) with faecal incontinence in the current study underwent local excision before chemoradiation. Only 12.0% in the surgery group showed T3 or T4 cancer (T1: 40.0%) compared with 35.0% in the non-surgery group (T1: 12.5%) - making a direct influence of the tumour size highly unlikely whereas surgery-related morbidity has to be considered when judging late gastrointestinal toxicity after chemoradiation. The current study has a number of limitations the most considerable being its retrospective nature. Disparities in patient distribution and a variable length of follow-up should be mentioned. On the other hand median follow-up of 41.4 months and the large cohort of 105 patients compared with similar reports is one of the strengths of our study. The results suggest that using IMRT technique severe acute toxicity of skin and gastrointestinal tract can be reduced while maintaining satisfactory results in disease control. However further analysis should be done evaluating the role of HPV and cetuximab regarding treatment outcome.

## Conclusion

This analysis demonstrates that concurrent chemotherapy and radiotherapy is the preferred standard of care for anal cancer patients. The use of IMRT/Tomotherapy is associated with less severe acute skin and gastrointestinal toxicity, but no benefit could be determined regarding OS, PFS, LC and CFS. Further analysis is necessary to identify additional markers in order to improve local control and PFS by escalating cancer treatment for selected patients.

## Competing interests

The authors declare that they have no competing interests.

## Authors’ contributions

SAK was the main investigator who collected and evaluated the clinical information. AS conducted the statistical analysis and critically evaluated the paper. MFH, DK and CS assisted the main author in data collection and revising the final draft. KK built the database for collecting the clinical information and AKS supported the statistical analysis. The paper was critically evaluated by KH and JD. FS designed and supervised the retrospective analysis. All authors read and approved the final version of the paper.
